# Analysis of Different Classification Techniques for Two-Class Functional Near-Infrared Spectroscopy-Based Brain-Computer Interface

**DOI:** 10.1155/2016/5480760

**Published:** 2016-09-20

**Authors:** Noman Naseer, Nauman Khalid Qureshi, Farzan Majeed Noori, Keum-Shik Hong

**Affiliations:** ^1^Department of Mechatronics Engineering, Air University, Sector E-9, Islamabad 44000, Pakistan; ^2^School of Mechanical Engineering and Department of Cogno-Mechatronics Engineering, Pusan National University, Busan 46241, Republic of Korea

## Abstract

We analyse and compare the classification accuracies of six different classifiers for a two-class mental task (mental arithmetic and rest) using functional near-infrared spectroscopy (fNIRS) signals. The signals of the mental arithmetic and rest tasks from the prefrontal cortex region of the brain for seven healthy subjects were acquired using a multichannel continuous-wave imaging system. After removal of the physiological noises, six features were extracted from the oxygenated hemoglobin (HbO) signals. Two- and three-dimensional combinations of those features were used for classification of mental tasks. In the classification, six different modalities, linear discriminant analysis (LDA), quadratic discriminant analysis (QDA), *k*-nearest neighbour (*k*NN), the Naïve Bayes approach, support vector machine (SVM), and artificial neural networks (ANN), were utilized. With these classifiers, the average classification accuracies among the seven subjects for the 2- and 3-dimensional combinations of features were 71.6, 90.0, 69.7, 89.8, 89.5, and 91.4% and 79.6, 95.2, 64.5, 94.8, 95.2, and 96.3%, respectively. ANN showed the maximum classification accuracies: 91.4 and 96.3%. In order to validate the results, a statistical significance test was performed, which confirmed that the* p *values were statistically significant relative to all of the other classifiers (*p* < 0.005) using HbO signals.

## 1. Introduction

Brain-computer interface- (BCI-) based systems provide a direct communication pathway between the brain and external devices without the need for any muscular movements [1]. BCI systems are based on two different approaches, namely, invasive and noninvasive. In invasive BCI systems, for the purpose of fine-quality brain-signal acquisition, electrodes are directly implanted into the brain, which entails high-risk surgery [2–4]. Noninvasive BCI systems, contrastingly, do not require any type of surgery, on which basis they often are preferred over invasive methods. In noninvasive BCI systems, different modalities—electroencephalography (EEG) [5–9], functional magnetic resonance imaging (fMRI) [10–12], and functional near-infrared spectroscopy (fNIRS) [7, 13–21]—have been used to acquire high-quality brain signals.

Although fMRI and EEG have shown positive developments for rehabilitation of patients suffering from different motor disabilities, for example, amyotrophic lateral sclerosis (ALS), locked-in syndrome (LIS), and other physical disabilities, fMRI machines are quite expensive as well as heavy, rendering them infeasible for the purposes of portable BCI systems [22]. More recently, alternative fNIRS-based BCI systems have been widely used due to their well-balanced spatial and temporal resolution, safety, ease of use (portability), and less susceptibility to gross electrophysiological artifacts caused by eye blinks, eyeball movements, and muscle activity [23]. Indeed, over the past few decades, fNIRS-based BCI systems have shown promising results in becoming an effective medium of communication for patients with disabilities [18].

Near-infrared spectroscopy (NIRS) functions by utilizing the near-infrared (NI) spectrum of light (wavelength 600~1000 nm) to measure the hemodynamic response represented by oxygenated hemoglobin (HbO) and deoxygenated hemoglobin (HbR), after which the modified Beer-Lambert law is used to determine the changes in the HbO and HbR concentrations (Δ*c*_HbO_(*t*) and Δ*c*_HbR_(*t*), resp.) [24–28]. Jobsis first introduced, in 1977, the principal of near-infrared spectroscopy [29], which entails the use of emitters and detectors separated by a distance of 3~4 cm. The distance is critical, as a small distance (1 cm) contains only a skin-layer contribution, while a large distance (5 cm) can result in low-quality and undesirable signals [23].

In fNIRS-based BCI studies, various mental tasks like motor imagery [15, 16], music imagery [17, 30–32], mental arithmetic (MA) tasks [17, 33, 34], object rotation [34–37], and others [38–41] have been used to acquire maximum classification accuracies that facilitate communication with patients suffering from LIS and ALS. In an fNIRS-based BCI system, the prefrontal cortex of the brain plays an important role in the acquisition of fine signals, for two specific reasons: Usually, it is not involved in motor disabilities, and its hair-free region enhances signal strength and penetration depth [24]. After acquiring brain signals using an fNIRS-based BCI system, the first step is to eliminate physiological noises using different kinds of filters [42], the next step is to extract the features from the signals, and the final step is to apply classification techniques to acquire the maximum accuracy for the specified task.

In recent decades, various classification schemes have been used in the fNIRS-based BCI area to classify different mental tasks and, thus, acquire maximum classification accuracies, thereby improving the quality and effectiveness of communication with patients suffering afflictions such as ALS and LIS [30, 33, 34, 43–45]. In this study, we acquired mental arithmetic (MA) task versus rest signals from the prefrontal cortex of the brain, after which we removed the signals' physiological noises using the 4th-order Butterworth band-pass filter [18, 19, 46]. Subsequently, those filtered signals were utilized to calculate the different combinations of the statistical properties of the time-domain signals. Then, after obtaining the features, we employed, to acquire maximum classification accuracies across all of the subjects using Δ*c*_HbO_(*t*) signals, different types of classifiers, that is, linear discriminant analysis (LDA), quadratic discriminant analysis (QDA), *k*-nearest neighbour (*k*NN), Naïve Bayes, support vector machine (SVM), and artificial neural networks (ANN). By using 2-dimensional Δ*c*_HbO_(*t*) feature combinations with those classifiers, the classification accuracies were 71.6 ± 1.1, 90.0 ± 1.3, 69.7 ± 0.5, 89.8 ± 1.4, 89.5 ± 1, and 91.4 ± 0.8, respectively, and using the 3-dimensional feature combinations, the classification accuracies were 79.6 ± 1.5, 95.2 ± 1, 64.5 ± 0.3, 94.8 ± 1.2, 95.2 ± 0.7, and 96.3 ± 0.3, respectively.

## 2. Materials and Methods

### 2.1. Subjects

Seven healthy subjects participated in the experiment. All of them had normal vision and no history of any physical, mental, or psychological disorder. The experiments were conducted in accordance with the latest Declaration of Helsinki, and verbal consent was obtained from all of the subjects after explaining the experimental paradigm.

### 2.2. Experimental Paradigm

**Figure 1:**
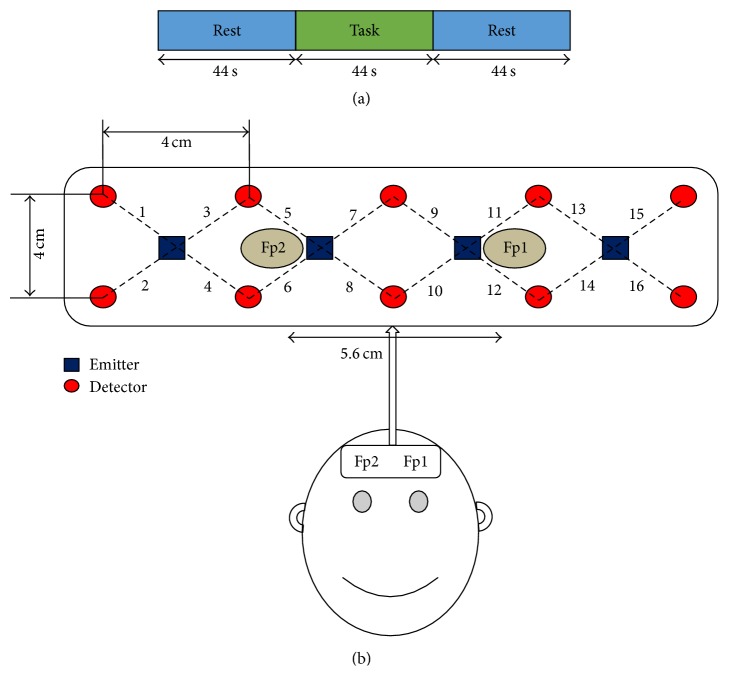
(a) Schematic of the experimental paradigm: the blue blocks represent the 44 s rest periods at the beginning and at the end; the second, green block represents the 44 s mental arithmetic task; (b) optode placement and channel location on the prefrontal cortex. Fp1 and Fp2 are the reference points of the international 10-20 system.

The subjects were seated in a quiet room on a comfortable chair in front of a computer monitor. They were asked to relax and to restrict their motor motions before the start of the experimental paradigm. The subjects were asked to rest and then to perform a mental arithmetic task, as shown in Figure 1(a). Specifically, each subject first rested for 44 s to adjust the baseline correction of the signals, and then he/she performed a mental arithmetic task for 44 s, of which paradigm was repeated five times. The total length of the experiment was 440 s for each subject. The 44 s task-rest periods are rather longer than the conventionally used 20 or 30 s task-rest periods [47–51]. The reason for using a longer duration was to get more data to extract statistical features for the purpose of training the classifiers. Of course, the statistical features are more reliable if the number of data points is larger. Since the main objective of this work was to determine the best performing classifier, training with the reliable and large amount of data was desirable. In the mental arithmetic task, the subjects performed a mental calculation consisting of the subtraction of a two-digit number (10~20) from a three-digit number with successive subtraction of another two-digit number from the result of the initial subtraction (e.g., 300 − 14, 286 − 11, and 275 − 16) [19, 43, 52].

### 2.3. Optodes Placement

A total of 4 emitters and 10 detectors were positioned on the prefrontal cortex for the detection of mental arithmetic and rest signals, of which configuration included 16 channels. In fNIRS-based BCI systems, the prefrontal cortex is the brain region most widely used, as the hairlessness incurs fewer and less slippage-relatedmotion artifacts and signal attenuation, respectively. The distance between the emitter and the detector plays an important role in the acquisition of fine-quality signals and the obtainment of maximum information therefrom [53]. Usually in fNIRS-based BCI systems, the emitter-to-detector distance is 3~4 cm [54]; in our research, the distance was set to 2.8 cm, as shown in Figure 1(b).

### 2.4. Signal Acquisition

A multichannel continuous-wave system (DYNOT: DYnamic Near-infrared Optical Tomography; two wavelengths: 760 and 830 nm; sampling rate: 1.81 Hz) obtained from NIRx Medical Technologies was used for the detection of brain activity. The near-infrared (NIR) light has been transmitted to the scalp from the source with the above-specified wavelength and then scattered through the cortical region of the brain where chromophores of HbO and HbR are present, which absorb some of the NIR light, the rest of which has been detected by the detectors.

### 2.5. Signal Processing

The modified Beer-Lambert law (MBLL) is used to calculate the concentration changes of HbO and HbR (Δ*c*_HbO_(*t*) and Δ*c*_HbR_(*t*)) in the microvessels of the cortex:

(1) ΔcHbOtΔcHbRt=1l×dαHbOλ1αHbRλ1αHbOλ2αHbRλ2−1ΔAt,λ1ΔAt,λ2,

where Δ*A*(*t*; *λ*_*j*_) (*j* = 1,2) is the absorbance (optical density) measured at two points of wavelength *λ*_*j*_, *a*_HbX_(*λ*_*j*_) is the extinction coefficient of HbX (i.e., HbO and HbR) in *µ*M^−1^ mm^−1^, *d* is the differential path length factor (DPF), and *l* is the emitter-detector distance (in millimetres). The signals obtained after conversion to Δ*c*_HbX_(*t*) contain physiological noises; so, we used a notch filter with band-reject ranges of 1~1.2 Hz, 0.3~0.4 Hz, and below 0.01 Hz to minimize the effects of such heartbeat-, respiration-, and Mayer-wave-related noises, respectively.

### 2.6. Feature Extraction

**Figure 2:**
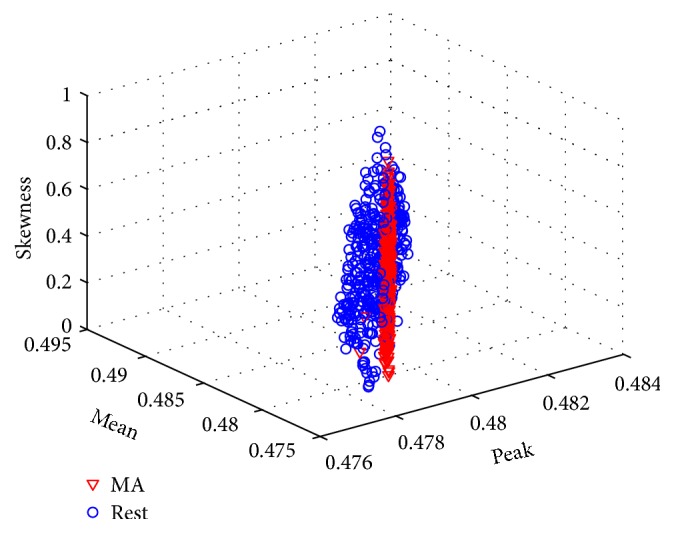
3D scatter plot of the signal mean, signal peak, and signal skewness values of HbO (subject 2).

In this study, we used the following statistical properties of time-domain signals as features: signal mean [18, 36, 45, 52, 55, 56], signal peak [33, 45, 57], signal slope [18, 58], signal variance [45, 59], signal kurtosis [45, 59], and signal skewness [45, 59]. Two- and three-dimensional combinations of those features were used for classification of the signals extracted from Δ*c*_HbO_(*t*). These features were calculated across all 16 channels spatially during the entire task and rest periods. All the features were normalized between 0 and 1 by the following equation [42]:

(2) $$\begin{array}{c} x^{\prime }=\frac{x-\min\left(x\right)}{\max\left(x\right)-\min\left(x\right)}, \end{array}$$

where *x*′ represents the feature values rescaled between 0 and 1, *x* ∈ *R* ^*n*^ are the original values of the features, and max⁡(*x*) and min⁡(*x*) represent the largest and smallest values, respectively.  Figure 2 shows the 3D feature space of the mental arithmetic and rest tasks for mean, speak, and skewness.

### 2.7. Classification

#### 2.7.1. Linear Discriminant Analysis

LDA has been most frequently used for pattern recognition in fNIRS-based BCI systems, thanks to its low computational cost and high speed [46, 55, 60–62]. Basically, LDA finds the projection to a line such that the samples from the classes are well separated from each other, thus achieving its main objective, dimensionality reduction. LDA does this, specifically, by maximizing the ratio of between-class variance and minimizing the ratio of within-class variance. The Matlab® command “classify linear” was used with 10-fold cross-validation to extract the classification performance.

#### 2.7.2. Quadratic Discriminant Analysis

QDA, likewise, maximizes the ratio of between-class variance and minimizes the ratio of within-class variance; however, it also allows quadratic decision boundaries between classes, thereby enabling the classifier to perform more effectively and enhancing classification accuracy [17, 63]. The Matlab® command “classify quadratic” was used with 10-fold cross-validation to extract the classification performance. In the present work, normal LDA and QDA, that is, without shrinkage or regularization, are used.

#### 2.7.3. *k*-Nearest Neighbour

*k*NN is the simplest classification technique used in fNIRS-based BCI systems for machine-learning algorithms [64]. The *k*NN algorithm works by determining which of the points from the training data are close enough to be considered when selecting the class to predict for a new observation. In the present research, the value of *k* was set to 1 in order to allow for the closest training samples of the class. The Matlab® command “*k*NN classify” was used with 10-fold cross-validation to extract the classification performance.

#### 2.7.4. Naïve Bayes Classifier

In addition to LDA, QDA, and *k*NN, the Naïve Bayes approach was also implemented in our study, due to its simplicity and transparency in machine-learning modalities. This approach is fundamentally based on the Bayes theorem with assumptions of strong independence among the features [65, 66]:

(3) $$\begin{array}{c} P\left(\;c\;|\;x\;\right)=\frac{P\left(x\;|\;c\right)P\left(c\right)}{P\left(x\right)}, \end{array}$$

where *P*(*c*∣*x*) is the feature probability of the class (target) of a given feature, *P*(*c*) is the prior probability of the class, *P*(*x*∣*c*) is the likelihood which is the probability of feature given class, and *P*(*x*) is the prior probability of the feature.

#### 2.7.5. Support Vector Machine

SVM is a widely employed classification modality in fNIRS-based BCI systems due to its high classification performance, relatively good scalability to high-dimensional data, and explicit control of errors [19, 34, 44, 59, 67, 68]. The main idea of SVM is to create the hyperplanes that maximize the margins between the classes that can be obtained by minimizing the cost function and, thereby, enable maximum classification accuracy. The vectors that represent the hyperplanes are known as support vectors. The optimal solution *r* ^*∗*^ that maximizes the distance between the hyperplane and the nearest training point(s) can be obtained by minimizing the cost function:

(4) Minimize 12w2+C∑i=1nξiSubject  to yiwTxi+b3≥1−ξi,ξi≥0,

where *w* ^*T*^, *x*_*i*_ ∈ *R* ^2^ and *b* ∈ *R* ^1^, ‖*w*‖^2^ = *w* ^*T*^ *w*, *C* is the trade-off parameter between error and margin,  *ξ*_*i*_ is the measure of training data, and *y*_*i*_ is the class label for the *i*th sample. The main advantage of SVM is that it can be used as both a linear and a nonlinear classifier. In order to make SVM a nonlinear classifier, one of various types of kernel functions (i.e., polynomial, radial basis, and sigmoid functions) can be used. In our present research, we utilized a third-degree polynomial kernel function with *C* = 0.5. Tenfold cross-validation was then used to estimate the classification accuracies. The reason for using nonlinear SVM is that it has been shown to yield better classification accuracies than the linear classifiers [19].

#### 2.7.6. Artificial Neural Networks

ANN is a classification technique widely used for deep machine-learning and pattern recognition in fNIRS-based BCI system [35, 69, 70]. The ANN classification modality plays an important role in the rehabilitation of patients suffering from afflictions such as ALS and LIS by decoding useful information. In our research, we used a three-layer perceptron consisting of an input, a hidden layer, and an output. The numbers of hidden neurons are specified by the following equation:

(5) $$\begin{array}{c} H=\left[\;\left(\;N+M\;\right)d\;\right], \end{array}$$

where *N* is the number of input neurons, *M* is the number of output neurons, and *d* is a constant with *d* ∈ (0,1]. For ANN classifier, the Matlab toolbox was used with 10 hidden neurons, 70% of the total data was used for training, 15% data was used for validation (measure of network generalization), and 15% data was used for testing (independent measure of network performance during and after training) [71].

## 3. Results and Discussion

In this study, we analyse and compare the performance of LDA, QDA, *k*NN, Naïve Bayes, SVM, and ANN classifiers in order to determine the best classifier for fNIRS-based BCI system using mental arithmetic tasks and rest. The classification accuracies for mental arithmetic task and rest were calculated for all possible 2- and 3-feature combinations of six different features. The extracted features include the signal mean, signal peak, signal skewness, signal slope, signal variance, and signal kurtosis. These features are calculated for the whole task and rest periods. It was found that the presence of signal mean and signal peak in both 2- and 3-feature combinations yielded maximum classification accuracies. This finding is an endorsement to our previous finding in [21].

**Figure 3:**
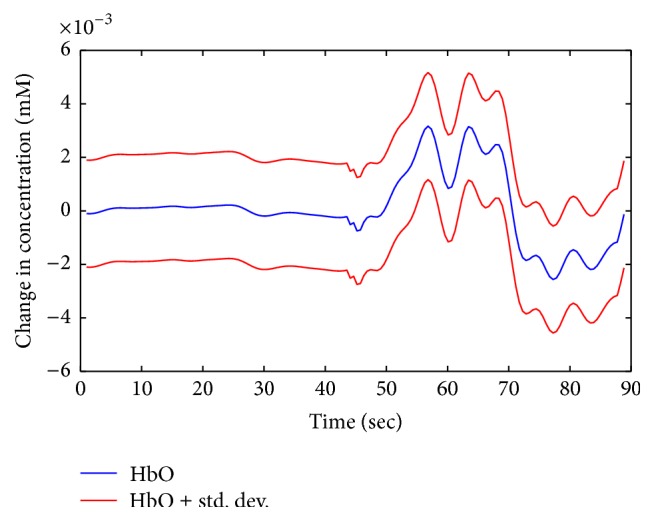
The averaged HbO and standard deviation (subject 2) for mental arithmetic and rest.

Tables 1, 2, 3, 4, 5, and 6 show the classification accuracies among all of the subjects for the respective classifiers. Those accuracies were extracted from 2-dimensional combinations of features derived from Δ*c*_HbO_(*t*) signals. The average classification accuracies of the LDA, QDA, *k*NN, Naïve Bayes, SVM, and ANN classifiers for the 2-dimensional feature combinations were 71.6 ± 1.1, 90.0 ± 1.3, 69.7 ± 0.5, 89.8 ± 1.4, 89.5 ± 1, and 91.4 ± 0.8, respectively. To further examine the performances of the classifiers used in our study, we also employed 3-dimensional combinations of features and extracted the corresponding classification accuracies, which were 79.6 ± 1.5, 95.2 ± 1.0, 64.5 ± 0.3, 94.8 ± 1.2, 95.2 ± 0.7, and 96.3 ± 0.3, respectively. In both (2- and 3-dimensional) cases, it was found that the ANN classifier has the highest classification accuracies: 91.4 and 96.3% for mental arithmetic task and rest.  Figure 3 shows the averaged HbO and standard deviation for mental arithmetic and rest task. Tables 7 and 8 provide the comparison of all classifiers—in terms of average classification accuracies, precision, and recall—across all subjects for 2- and 3-feature combination, respectively. In order to validate that our ANN classification accuracies were statistically discriminant, we applied Student's *t*-test. The *p* values obtained using the ANN values versus those of all of the other classifiers were less than 0.05 for all of the Δ*c*_HbO_(*t*) signals, thus establishing the statistical significance of ANN's performance.

**Figure 4:**
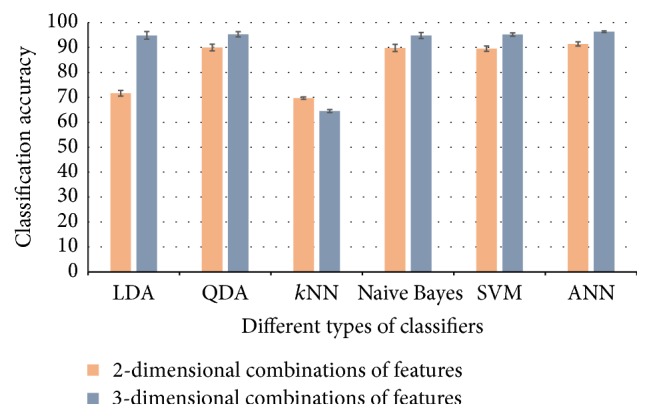
Classification accuracies using different types of classifiers from 2- and 3-dimensional combinations of features of Δ*c*_HbO_(*t*) signals across all subjects.

Several previous studies have used multiple types of classifiers to extract the classification accuracies for fNIRS-based BCI system. For example, Naseer et al. [19] have used LDA and SVM to acquire the classification accuracies for a two-class BCI system, the classification accuracies were 74.2 and 82.1% respectively. Moreover, Khan and Hong [72] used LDA and SVM classifiers for a two-class BCI system; the classification accuracies were 84.6 and 85.8%. In the present study, the six different classifiers were used to obtain the highest average classification accuracies for a two-class (metal arithmetic and rest) BCI system. The ANN classifier showed the maximum average classification accuracies 91.4 and 96.3% for 2- and 3-dimensional combinations of features derived from Δ*c*_HbO_(*t*) signals, respectively.  Figure 4 plots the average accuracies of all of the classifiers used in this study for 2- and 3-dimensional combinations of features derived from Δ*c*_HbO_(*t*) signals. One of the limitations of the current work is the small number of subjects. Analysis with large number of subject can yield to well establishing of the results. Another limitation of our current study is that we used the two-class mental task (metal arithmetic and rest) for an fNIRS-based BCI system. For three- and more-class BCI problems, other classifier modalities might yield better results. In any case, further research entailing the examination of the results of multiple mental task classifications using different types of classification modalities for fNIRS-based BCI systems is required. Furthermore, inherent delay in fNIRS systems can be removed by detection of initial dips to improve BCI accuracy [73].

## 4. Conclusion

In this study, we examined the effects of using different classification modalities for the classification of a two-class functional near-infrared spectroscopy- (fNIRS-) based brain-computer interface (BCI) according to a mental arithmetic task and rest experimental paradigm. It was shown that ANN has the highest classification accuracies among the classification modalities used in this study for both 2- and 3-dimensional feature sets derived from Δ*c*_HbO_(*t*) signals across seven subjects. The results of this study represent a significant step forward in the on-going improvement of the classification accuracies of fNIRS-based BCI systems.

## Figures and Tables

**Table 1:** Classification accuracy using LDA among all subjects.

Feature combination	S1	S2	S3	S4	S5	S6	S7
Mean & slope	53.2	49.2	50.1	58.3	59.8	55.8	59.6
Mean & peak	94.5	96.7	90.3	92.0	91.1	92.2	94.9
Mean & variance	86.8	87.6	81.7	82.9	82.8	76.4	83.4
Slope & peak	87.3	83.6	80.8	85.9	83.8	83.6	81.2
Slope & variance	87.5	88.3	83.2	82.6	81.5	76.4	79.9
Peak & variance	89.7	89.8	83.7	87.5	87.3	83.7	81.2
Peak & skewness	89.1	83.6	80.4	86.5	81.6	83.2	81.2
Mean & skewness	49.6	50.6	48.6	53.3	53.6	53.5	50.1
Slope & skewness	50.5	51.2	50.3	53.8	54.0	53.1	50.9
Kurtosis & skewness	47.7	51.2	50.6	53.2	50.4	53.8	51.6
Variance & skewness	88.0	89.0	82.3	83.4	81.4	78.2	84.4
Peak & kurtosis	86.8	82.4	80.9	85.9	83.9	82.6	81.2
Mean & kurtosis	47.4	49.7	52.2	54.8	50.4	52.1	48.6
Slope & kurtosis	45.7	46.2	54.3	54.6	52.1	50.1	47.7
Variance & kurtosis	87.6	88.5	82.1	83.2	82.4	82.2	86.3

**Table 2:** Classification accuracy using QDA among all subjects.

Feature combination	S1	S2	S3	S4	S5	S6	S7
Mean & slope	95.5	96.5	95.5	96.6	96.0	95.4	96.9
Mean & peak	97.0	98.4	97.7	98.2	97.4	98.4	98.4
Mean & variance	97.4	98.0	96.0	96.5	96.1	95.4	97.2
Slope & peak	94.4	95.0	88.5	95.7	95.1	93.6	96.1
Slope & variance	98.0	98.2	94.0	95.5	94.4	93.0	95.0
Peak & variance	97.0	98.1	93.7	97.4	96.0	94.1	96.9
Peak & skewness	90.6	89.8	83.8	92.6	89.7	88.8	86.8
Mean & skewness	91.0	91.5	90.5	91.8	89.1	87.6	93.2
Slope & skewness	89.3	89.7	84.2	88.0	90.0	86.6	91.2
Kurtosis & skewness	48.3	53.7	51.4	52.8	50.2	56.1	52.1
Variance & skewness	97.5	97.9	90.7	95.6	94.7	90.7	92.1
Peak & kurtosis	89.6	88.2	82.7	92.1	88.1	88.2	86.2
Mean & kurtosis	89.2	91.1	88.8	90.8	88.8	87.5	93.9
Slope & kurtosis	89.5	89.6	84.3	87.7	89.8	86.6	91.0
Variance & kurtosis	97.6	97.9	90.6	95.4	94.4	90.6	91.7

**Table 3:** Classification accuracy using *k*NN among all subjects.

Feature combination	S1	S2	S3	S4	S5	S6	S7
Mean & slope	94.4	94.6	95.5	95.1	94.4	93.5	94.6
Mean & peak	95.9	96.2	97.2	97.6	97.6	97.1	96.7
Mean & variance	89.1	92.6	91.5	91.3	90.7	89.6	94.0
Slope & peak	92.5	91.1	91.7	95.9	93.1	93.6	92.6
Slope & variance	95.0	95.1	94.0	93.7	93.2	92.5	93.6
Peak & variance	88.2	88.7	86.8	93.5	88.6	90.2	86.3
Peak & skewness	64.4	66.9	64.5	65.4	62.5	58.0	61.0
Mean & skewness	53.7	57.7	55.6	54.1	53.7	52.2	58.6
Slope & skewness	50.8	47.8	51.4	50.1	49.9	51.3	53.8
Kurtosis & skewness	47.3	50.7	55.0	51.3	54.3	60.1	54.8
Variance & skewness	50.6	48.1	51.2	49.7	49.3	51.4	54.7
Peak & kurtosis	65.4	63.4	59.2	65.7	62.7	55.0	60.7
Mean & kurtosis	53.5	55.0	52.9	52.1	52.2	51.3	53.7
Slope & kurtosis	52.1	49.8	50.7	48.6	50.9	48.8	50.7
Variance & kurtosis	51.9	50.4	50.6	48.9	50.6	48.8	50.7

**Table 4:** Classification accuracy using Naïve Bayes among all subjects.

Feature combination	S1	S2	S3	S4	S5	S6	S7
Mean & slope	95.6	96.9	95.2	96.1	96.4	94.7	96.9
Mean & peak	96.5	98.1	97.1	97.9	96.0	97.9	98.0
Mean & variance	97.5	98.1	96.0	96.6	96.4	95.7	97.1
Slope & peak	94.4	95.0	89.2	95.2	95.0	93.6	96.4
Slope & variance	98.0	98.0	92.0	95.2	94.9	92.0	94.4
Peak & variance	96.9	98.1	92.9	96.9	96.2	92.4	95.4
Peak & skewness	89.6	88.6	82.8	91.8	87.2	88.0	86.0
Mean & skewness	89.3	91.1	89.1	90.6	89.3	87.7	93.2
Slope & skewness	89.5	89.6	83.9	87.8	90.0	86.5	91.3
Kurtosis & skewness	51.1	50.8	51.1	52.7	51.8	55.8	51.9
Variance & skewness	97.7	97.9	90.3	95.6	94.7	90.6	92.1
Peak & kurtosis	89.6	88.2	82.8	92.0	87.1	88.2	86.2
Mean & kurtosis	89.1	91.2	88.8	90.7	89.0	87.6	93.9
Slope & kurtosis	89.6	89.6	83.9	87.7	89.6	85.8	91.2
Variance & kurtosis	97.6	97.9	90.5	95.4	94.5	90.6	92.0

**Table 5:** Classification accuracy using SVM among all subjects.

Feature combination	S1	S2	S3	S4	S5	S6	S7
Mean & slope	93.2	95.5	92.5	95.7	95.2	93.0	94.2
Mean & peak	97.0	98.5	98.5	98.7	97.7	98.5	98.7
Mean & variance	97.7	98.0	97.7	98.0	97.7	97.0	97.2
Slope & peak	92.7	92.7	88.9	96.0	94.2	90.2	91.7
Slope & variance	98.0	98.0	97.5	97.7	96.2	95.2	96.0
Peak & variance	98.0	98.7	97.5	97.5	98.0	96.7	98.5
Peak & skewness	92.7	85.2	83.9	93.7	90.5	86.9	83.4
Mean & skewness	89.5	88.9	84.9	88.2	88.4	86.2	87.7
Slope & skewness	82.4	86.4	84.9	84.7	86.4	82.7	86.7
Kurtosis & skewness	54.5	54.8	51.3	52.5	54.8	52.8	50.8
Variance & skewness	98.0	98.0	97.0	97.5	96.2	95.7	96.2
Peak & kurtosis	90.2	83.4	81.2	94.0	86.2	84.2	83.2
Mean & kurtosis	87.7	89.2	85.4	89.2	88.9	84.4	87.7
Slope & kurtosis	82.7	86.7	83.9	83.2	86.4	81.2	86.2
Variance & kurtosis	98.5	98.5	97.2	97.7	96.7	95.7	96.7

**Table 6:** Classification accuracy using ANN among all subjects.

Feature combination	S1	S2	S3	S4	S5	S6	S7
Mean & slope	95.6	97.4	94.9	97.1	96.6	96.0	95.6
Mean & peak	96.9	98.5	98.1	98.7	97.9	98.4	98.4
Mean & variance	98.0	98.4	98.1	97.7	98.2	96.4	98.2
Slope & peak	95.4	92.3	95.9	96.1	95.5	90.2	92.7
Slope & variance	97.9	98.4	97.7	98.2	98.0	97.0	98.0
Peak & variance	98.1	97.9	97.4	97.2	98.0	97.4	98.4
Peak & skewness	92.8	89.0	84.2	94.1	91.6	88.5	84.7
Mean & skewness	92.0	94.0	90.6	92.8	89.8	88.7	92.7
Slope & skewness	90.8	88.0	88.2	90.7	90.0	91.6	90.6
Kurtosis & skewness	54.5	55.7	53.6	55.6	56.2	55.7	51.6
Variance & skewness	98.4	98.5	97.6	98.4	97.2	97.0	98.0
Peak & kurtosis	90.1	88.5	81.4	94.1	87.8	87.5	84.9
Mean & kurtosis	90.1	93.5	89.0	91.6	90.7	89.8	91.5
Slope & kurtosis	91.3	88.1	88.2	90.0	90.6	91.9	90.3
Variance & kurtosis	98.4	98.4	97.1	97.4	97.6	96.6	95.5

**Table 7:** Averaged values of the classification accuracies, precisions, and recalls of 2-feature combination across all subjects.

Classifiers	S1	S2	S3	S4	S5	S6	S7	Average
LDA								
Accuracy	72.74	72.49	70.09	73.20	71.74	70.44	70.80	71.6 ± 1.1
Precision	79.34	79.62	79.74	68.73	67.28	66.21	66.36	72.8 ± 6.2
Recall	66.50	65.28	58.30	83.45	80.65	81.43	78.70	73.5 ± 9.2

QDA								
Accuracy	90.78	91.57	87.49	91.12	89.98	88.83	90.57	90.1 ± 1.3
Precision	93.80	95.84	95.32	87.68	86.27	84.00	87.28	90.0 ± 4.4
Recall	89.63	88.80	79.53	96.50	93.65	96.82	93.65	91.2 ± 5.5

*k*NN								
Accuracy	69.63	69.87	69.85	70.19	69.58	68.89	70.44	69.8 ± 0.5
Precision	67.97	68.73	69.90	70.31	71.24	67.16	68.18	69.1 ± 1.3
Recall	70.81	72.14	72.31	68.21	74.53	66.32	68.38	70.4 ± 2.6

Naïve Bayes								
Accuracy	90.79	91.27	87.04	90.82	89.86	88.47	90.39	89.8 ± 1.4
Precision	88.52	88.01	81.73	95.69	94.88	95.93	95.48	91.5 ± 5.1
Recall	92.12	94.16	94.76	85.61	85.96	79.90	86.85	88.5 ± 5.0

SVM								
Accuracy	90.18	90.17	88.16	90.96	90.25	88.02	88.99	89.5 ± 1.0
Precision	87.04	95.84	95.32	87.68	86.27	84.00	87.28	89.1 ± 4.2
Recall	93.80	88.80	79.53	96.50	93.65	96.82	93.65	91.8 ± 5.5

ANN								
Accuracy	92.02	91.77	90.13	92.65	91.71	90.85	90.74	91.4 ± 0.3
Precision	94.47	89.73	93.20	90.73	86.68	88.03	87.65	90.1 ± 2.7
Recall	88.00	86.33	85.13	95.33	94.05	95.73	95.63	91.5 ± 4.4

**Table 8:** Averaged values of the classification accuracies, precisions, and recalls of 3-feature combinations across all subjects.

Classifiers	S1	S2	S3	S4	S5	S6	S7	Average
LDA								
Accuracy	81.24	81.76	78.41	80.54	79.68	77.88	77.78	79.6 ± 1.5
Precision	89.9	89.21	89.66	75.09	74.21	72.37	72.53	80.4 ± 7.8
Recall	73.94	73.98	65.79	91.33	90.09	89.88	87.48	81.8 ± 9.5

QDA								
Accuracy	95.84	96.58	93.49	95.96	95.11	94.12	95.93	95.2 ± 1
Precision	97.26	98.67	98.67	93.79	92.34	90.8	93.85	95.1 ± 2.9
Recall	94.41	94.1	88.19	98.71	98.64	98.8	98.66	95.9 ± 3.7

*k*NN								
Accuracy	63.92	65.32	64.85	64.78	64.59	63.55	65.11	64.5 ± 0.3
Precision	63.22	62.21	63.85	64.13	65.85	61.04	61.07	63.1 ± 1.6
Recall	65.28	67.56	66.15	61.67	69.49	60.13	61.54	64.6 ± 3.2

Naïve Bayes								
Accuracy	95.58	96.34	92.77	95.53	94.72	93.47	95.55	94.8 ± 1.2
Precision	94.45	94.22	88.46	98.73	98.35	98.64	98.68	95.9 ± 3.6
Recall	96.98	99.12	98.95	92.22	90.89	88.1	92.29	94.1 ± 3.9

SVM								
Accuracy	95.79	95.75	94.21	95.97	95.58	94.47	94.85	95.2 ± 0.7
Precision	93.22	99.03	98.67	93.79	92.34	90.8	93.85	94.5 ± 2.9
Recall	99.12	94.1	88.19	98.71	98.64	98.8	98.66	96.6 ± 3.8

ANN								
Accuracy	96.48	96.49	95.78	96.87	96.4	95.97	96.41	96.3 ± 0.3
Precision	93.9	94.4	98.1	95.05	93.26	91.59	93.71	94.3 ± 1.9
Recall	91.9	92.8	91.55	93.64	98.39	98.46	98.67	95.1 ± 3.0
